# Pygo2 functions as a prognostic factor for glioma due to its up-regulation of H3K4me3 and promotion of MLL1/MLL2 complex recruitment

**DOI:** 10.1038/srep22066

**Published:** 2016-02-23

**Authors:** Cefan Zhou, Yi Zhang, Jun Dai, Mengzhou Zhou, Miao Liu, Yefu Wang, Xing-Zhen Chen, Jingfeng Tang

**Affiliations:** 1 Membrane Protein Disease and Cancer Research Center, Provincial Cooperative Innovation Center of Industrial Fermentation, College of Bioengineering, Hubei University of Technology, Wuhan, Hubei, 430068, China; 2 Neurology department, Renmin Hospital of Wuhan University, Wuhan, Hubei, 430060, China; 3 The State Key Laboratory of Virology, College of Life Sciences, Wuhan University, Wuhan, Hubei, 430072, China; 4 Membrane Protein Disease Research Group, Department of Physiology, Faculty of Medicine and Dentistry, University of Alberta, Edmonton, AB, Canada

## Abstract

Pygo2 has been discovered as an important Wnt signaling component contributing to the activation of Wnt-target gene transcription. In the present study, we discovered that Pygo2 mRNA and protein levels were up-regulated in the majority of (152/209) human brain glioma tissues and five glioma cell lines, and significantly correlated with the age, the WHO tumor classification and poor patient survival. The histone methyltransferase complex components (WDR5, Ash2, and menin, but not CXCC1 or NCOA6) were down-regulated at the promoter loci of Wnt target genes after Pygo2 knockdown, and this was accompanied by the down-regulation of Wnt/β-catenin pathway activity. Further, we demonstrated that the involvement of Pygo2 in the activation of the Wnt pathway in human glioma progression is through up-regulation of the H3K4me3 (but not H3K4me2) by promoting the recruitment of the histone methyltransferase MLL1/MLL2 complex to Wnt target gene promoters. Thus, our study provided evidence that Pygo2 functions as a novel prognostic marker and represents a potential therapeutic target.

The Wnt/β-catenin signaling pathway is a highly conservative signaling pathway that is involved in the process of evolution and is widely found in invertebrates and vertebrates. The canonical Wnt/β-catenin pathway plays a crucial role in early development including embryogenesis, organogenesis, tissue regeneration and other physiological processes^1^. Abnormal signal activation due to mutations in or abnormal expression of components of the pathway was known result in tumorigenesis^2^,^3^. The activation state of the canonical Wnt/β-catenin pathway is determined by the nuclear levels of the β-catenin protein^4^. The binding of secreted Wnt ligands to frizzled transmembrane receptors and low-density lipoprotein (LRP5/6) co-receptors initiates the disaggregation of the hetero-tetramer composed of axin, adenomatous polyposis coli, glycogen synthase kinase-3b and CKI, thereby inhibiting β-catenin phosphorylation and promoting its nuclear localization. Nuclear β-catenin binds to T-cell factor/lymphoid-enhancing factor (TCF/LEF), B-cell lymphoma-9 (Bcl-9) and Pygopus 2 (Pygo2), which initiates transcription of target genes involved in cell growth and proliferation, such as Cyclin D1, C-myc, CyclinA and CD44, which all play important roles in tumorigenesis, tumor progression and prognosis^5^,^6^,^7^,^8^.

There are two Pygopus genes: the paralogs Pygo1 and Pygo2. Pygo2 is more widely expressed^9^ and is considered to play a more important role while mice deficient for both Pygo1 and Pygo2 do not display an exacerbated phenotype than deficient for Pygo2 only^10^. Pygo2 has been reported to be over expressed in, and important for the growth of, several types of malignant tumors, including epithelial ovarian cancer and breast cancer^11^,^12^, reduced Pygo2 expression by gene knockdown inhibited cell proliferation and invasiveness in human glioblastoma U251 cells^13^. There is a highly conserved structure named the PHD domain with a Zn^2+^ coordinating finger in the C terminus of Pygo2^14^; previous studies have shown that PHD-containing proteins can act as protein code readers to link the chromatin remodeling complex to specific changes in gene transcription, as demonstrated for the Wnt/β-catenin target genes. However, the regulation of Pygo2 protein expression in malignancy remains poorly understood.

The catalysis of the histone tails plays a crucial role in regulating chromatin structure and controlling transcriptional activity. Histone H3 trimethylation at lysine 4 (H3K4me3) is associated with gene expression in eukaryotes^15^,^16^,^17^. The generation of mono-, di-, and tri-methylated histone is catalyzed by lysine methyl transferases family 2 (KMT2) members. This family of enzymes is found within a macromolecular complex known as COMPASS and is highly conserved from yeast to humans. There are seven members in the Set1/COMPASS complex in yeast, which was identified as the first H3K4 methylase. In mammalian cells, the complex bears five COMPASS family members (SET1, MLL1 and MLL2, and MLL3 and MLL4) that share five common components (ASH2L, RBBP5, DPY30, HCF1 and WDR5). Each COMPASS family member consists of a complex of specific subunits. In fact, SET1 complexes uniquely associate with WDR82 and CXXC1,while MLL1/MLL2 complexes associate with Menin and MLL3/4 complexes contain PTIP,PA-1, UTX, and NCOA6^18^.

Here, we investigated the Pygo2 expression profile in human brain glioma and found that the Pygo2 protein and mRNA were over expressed in the majority of patient glioma tumor tissues. The high expression level of Pygo2 indicated a high risk for brain glioma oncogenesis. We also confirmed that Pygo2 was located in the nucleus of glioma cells using immunofluorescence staining and immunohistochemistry. We demonstrated that the level of H3K4me3 was reduced using Pygo2-specific RNA interference knockdown technology, resulting in the inhibition of the activation of Wnt pathway target genes. These results showed an indispensable role for Pygo2 in the growth of the human brain glioma U-87MG and U251 cell lines. These finding suggest the efficacy of Pygo2 as a diagnostic and prognostic biomarker in patients with glioma.

## Results

### Over-expression of Pygo2 in primary glioma tissues and cell lines

**Figure 1:**
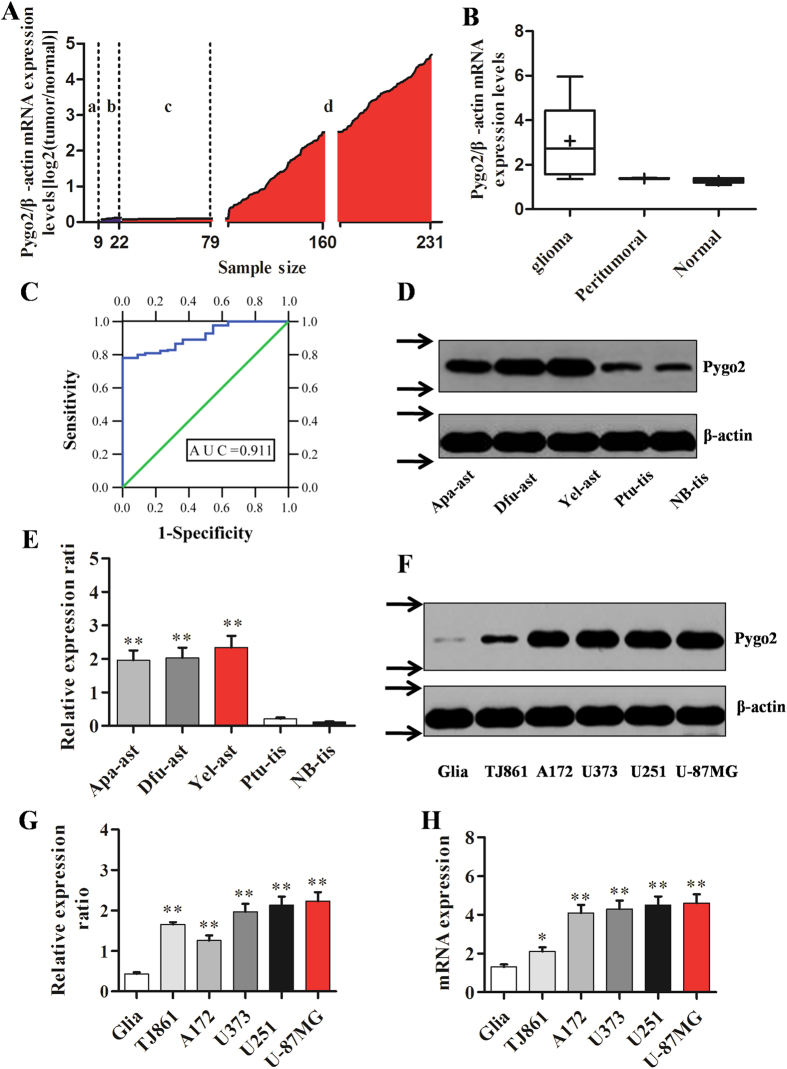
Expression of Pygo2 in primary glioma tissues and cell lines. (**A**) Pygo2 mRNA expression was examined by qRT-PCR in 231 human tissues composed of 9 normal tissues (a), 13 peritumoral tissues (b) and 209 human glioma tissues (c,d). Pygo2 expression was normalized to β-actin in each sample. (**B**) Box plot of human Pygo2 expression in 9 normal, 13 peritumoral and 209 glioma tissues. Boxes represent interquartile ranges, and the horizontal lines across each box indicate median values. (**C**) The ROC curve of Pygo2 expression in glioma patients from normal subjects. The Pygo2 expression levels made an area under the curve (AUC) value of 0.91, with a standard error of 0.02 and 95% confidence interval of 0.88–0.94, based on the method used by Delong *et al.* 1998. (**D**,**E**) The Pygo2 protein levels in anaplastic astrocytoma (Apa-ast), astrocytoma (Dfu-ast), astrocytoma (Yel-ast), peritumoral (Ptu-tis) tissues and Normal brain tissue (NB-tis, control). Statistical data were obtained from three independent measurements as mean ± SD. (**F**–**H**) The Pygo2 protein and mRNA levels in 5 Glioma cell lines, TJ861, A172, U373, U251, and U-87MG. Normal human microglia cells (Glia) were used as a control. Arrows (**→**) (panel **F**) indicate cropping lines. Full-length blots/gels are presented in Supplementary Figure 1 and were obtained under the same experimental conditions. Data were presented as mean ± SD. Two-tailed Student’s t-test was used. *P < 0.05 and **P < 0.01.

To investigate the abnormal Pygo2 expression in human glioma, QRT-PCR was first performed and the Pygo2 expression normalized to β-actin in each sample. As shown in Fig. 1A, Pygo2 mRNA expression in the majority (152 out of 209) of primary glioma tissue samples (d) was increased compared with that in normal tissues (n = 9) (a), while peritumoral tissues (n = 13) (b) showed no significant changes. The quantified Pygo2 mRNA levels were shown in Fig. 1B. These data showed that Pygo2 mRNA level was significantly increased in the glioma tissues (P < 0.01).

Our data are consistent with previous reports that Pygo2 mRNA expression is increased in several cancer models such as NSCLC, breast cancer and ovarian cancer, among others^19^,^20^,^21^. To assess a potential of Pygo2 as diagnostic and prognostic marker of glioma, we generated ROC curves and found that the Pygo2 mRNA level in glioma tissues substantially differs from that in control subjects, with an AUC value of 0.91 (Fig. 1C). Using the criterion value of 1.4, the sensitivity and specificity values were 0.78 and 1, respectively, to identify a patient with glioma, indicating that Pygo2 serves as an excellent glioma marker.

Western blot analysis was then used to examine the protein expression of Pygo2 in glioma tissues which were classified into anaplastic astrocytoma tissue, diffuse astrocytoma tissue, yellow astrocytoma tissue, and normal tissue (Fig. 1D,E). Up-regulation of Pygo2 was observed in anaplastic astrocytoma (Apa-tis), diffuse astrocytoma (Dfu-tis) and yellow astrocytoma (Yel-tis) when compared with peritumoral tissue (Ptu-tis) and normal brain tissue (NB-tis). We also used western blot and qRT-PCR analyses to examine the expression of Pygo2 and β-actin (as control) in five glioma cell lines, TJ861, A172, U373, U251 and U-87MG and found that Pygo2 protein and mRNA levels are both up-regulated as compared with normal human microglia primary cells (Glia) (Fig. 1F–H). These data underscore the importance of Pygo2 as a specific biomarker for the diagnosis of glioma. In addition, and since aberrant over-expression of Wnt components has been previously reported in human glioma^15^,^22^, our present result suggests that there may be a functional significance of the Pygo2 over-expression in the Wnt signaling in human glioma.

### Pygo2 over-expression correlates with glioma clinicopathological features and patient survival

We next investigated the correlation of the Pygo2 expression level with clinicopathological features. We found that over-expression of Pygo2 in human brain glioma significantly correlates with the age and the tumor grade by WHO, but not with the gender (Table 1). This result signifies that over-expression of Pygo2 occurred more frequently in the elderly (>50 years) and advanced tumors (P < 0.01).

**Figure 2:**
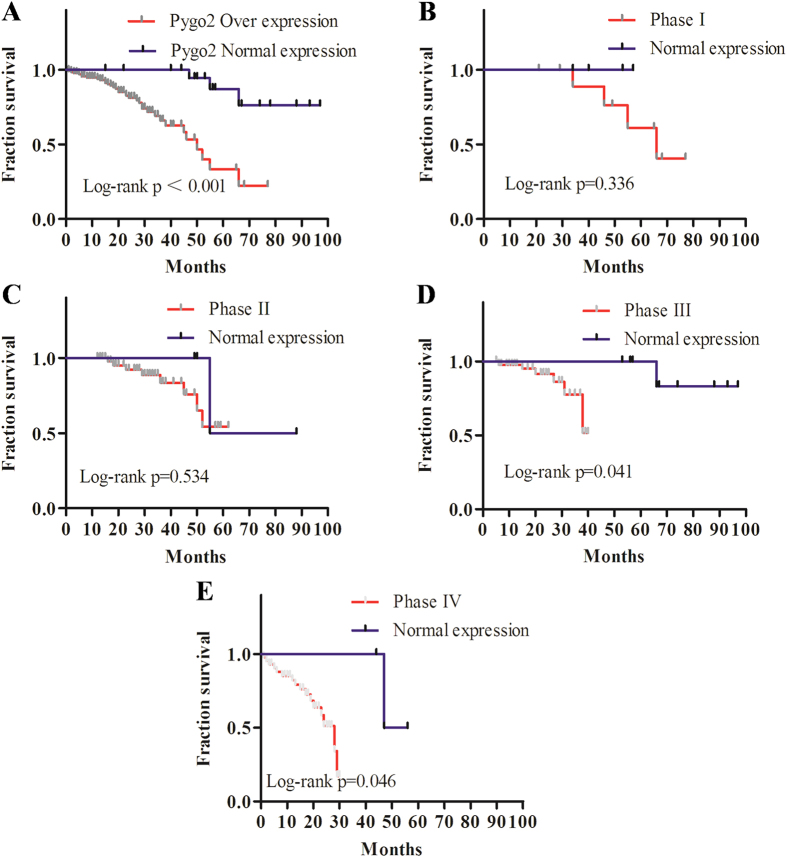
Correlation of Pygo2 expression with patient survival rate. (**A**) Kaplan-Meier survival curves obtained using 209 human brain glioma patients and 9 normal subjects. (**B–E**) Kaplan-Meier survival curves of Pygo2 over-expression were classified into phases I (**B**, n = 26), II (**C**, n = 77), III (**D**, n = 59) and IV (**E**, n = 47) based on the WHO guideline.

To further investigate whether the expression level of Pygo2 correlates with the survival of glioma patients, Kaplan-Meier analysis for the WHO tumor phases of 209 human brain glioma patients and 9 normal subjects was used (Fig. 2). We found that patients with Pygo2 over-expression have a poorer overall survival than those with Pygo2 normal expression (Fig. 2A). When patients with Pygo2 over-expression were further classified into four phases, those within phases III (n = 59, P = 0.041) and IV (n = 47, P = 0.046) showed poorer overall survival than those with Pygo2 normal expression, but those within phases I (n = 26, P = 0.34) and II (n = 77, P = 0.54) showed no significant difference compared with those with Pygo2 normal expression. Overall, our data showed that abnormal Pygo2 expression correlates with tumor progression and poor prognosis in human brain glioma.

### Nuclear localization of Pygo2 in glioma tissues and cell lines

**Figure 3:**
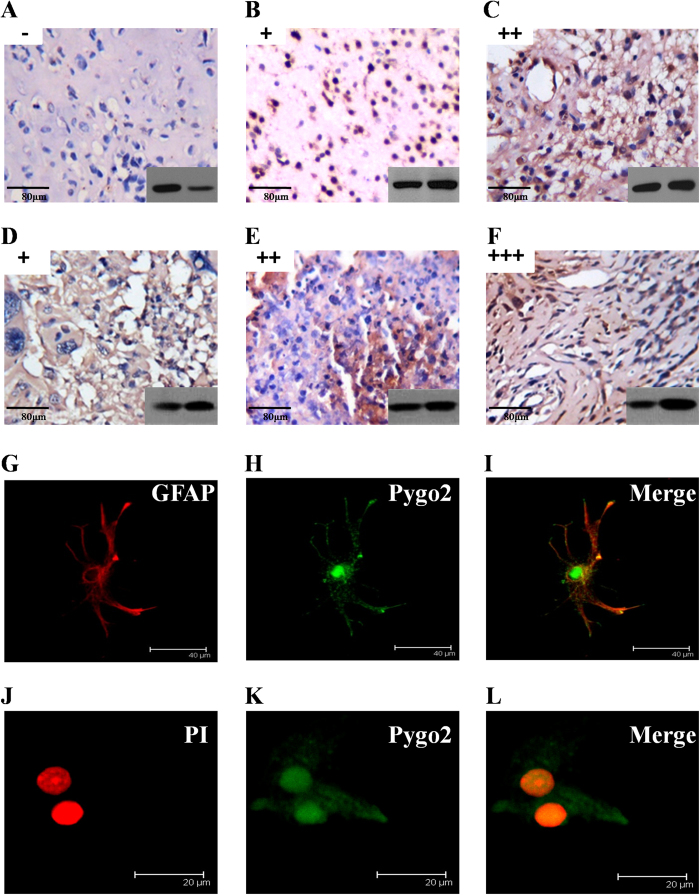
Nuclear localization of Pygo2 in glioma tissues and cell lines. (**A–F**) Pygo2 protein expression in glioma was classified by the intensity and frequency of tumor cell nuclei staining with Pygo2 antisera, and further classified as negative (−), weak (+, <70% nuclei staining), moderate (++, 70–90% nuclei staining), or strong +++, >90% nuclei staining). Negative (−) or weak (+) Pygo2 staining was detected in the nuclei of normal and peritumoral tissues (**A**). Weak (+) or moderate (++) Pygo2 nuclear staining was detected in astrocytoma and anaplastic astrocytoma (**B**,**C**). Weak (+) to strong (+++) Pygo2 nuclear staining was detected in glioblastoma multiforme (**D–F**). Original magnification was ×200. Bar, 80 μm. Western blotting images in the right bottom corner shows the Pygo2 expression levels. (**G–I**) Double staining for fibrillary acidic protein (GFAP)/red and Pygo2/green (astrocytes) in rat primary astrocytes. Pygo2 antisera were detected with fluorescein-conjugated secondary antibodies, and GFAP monoclonal antibodies detected with TRITC conjugated secondary antibodies. They were viewed under a fluorescence microscope. Bar 40 μm. (**J–L**) Double staining for Propidium Iodide/red and Pygo2/green (astrocytes) in rat primary astrocytes. Astrocytes were stained simultaneously with nuclear-specific Propidium Iodide, and Pygo2 antisera detected with fluorescein-conjugated secondary antibodies. Bar 20 μm.

To investigate the prevalence of Pygo2 expression in human brain glioma and normal tissues, we analyzed the *in situ* localization of Pygo2 in 231 tissues from archived surgical samples. The expression of Pygo2 was assessed semi-quantitatively based on the staining intensity and percentage of Pygo2 protein stained in both the nucleus and cytoplasm. Pygo2 was undetectable or barely detectable in the nucleus of normal or peritumoral tissues (Fig. 3A, “−”). In contrast, with the increase of glioma malignant potential, the nuclei of astrocytoma tissues were round, triangle or fusiform, and cells were moderately abnormal, with uneven distribution and a single or dual core, Pygo2 protein was mainly expressed in the nucleus and much less in the cytoplasm (Fig. 3B, “+”). Anaplastic astrocytoma showed higher cell density and mitotic activities than that in astrocytoma tissues (Fig. 3C, “++”) with a malignant potential between low potential malignancy astrocytoma and glioblastoma Multiforme. Pygo2 protein was strongly expressed in the nuclei of Glioblastoma tissues (Fig. 3D–F), which indicates endothelial cell hyperplasia, atypical proliferation, and increased karyokinesis. Overall, glioma from astrocytoma and glioblastoma Multiforme, but not normal or peritumoral tissues, had weak (“+”, 7.38%) to strong (“+++”, 29.53%) Pygo2 nuclear accumulation.

To further investigate the subcellular localization of endogenous Pygo2, we examined rat astrocytes in primary cultures by co-immunofluorescence with confocal microscopy. Endogenous Pygo2 protein was predominantly found in nuclei while it was weekly expressed in the cytoplasm (Fig. 3H–M). These observations are consistent with a role of Pygo2 as a nuclear protein in malignant glioma disease.

### Pygo2 knockdown suppresses the activity of the canonical Wnt signal pathway in glioma cell lines

**Figure 4:**
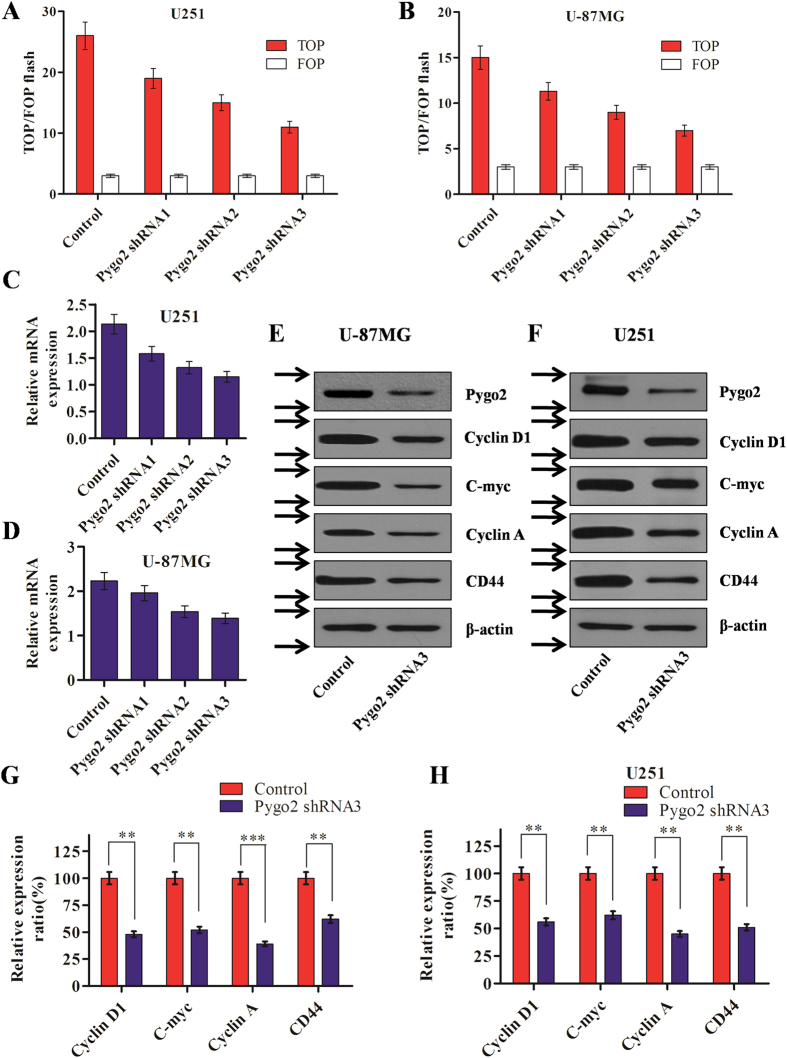
Effects of Pygo2 knockdown on the activity of the canonical Wnt signal pathway in glioma cell lines. (**A,B**) Effects of Pygo2 knockdown by shRNAs on TOP flash luciferase activity in human brain glioma U-87MG and U251 cell lines. Pygo2 shRNA1-3 and control (scr-shRNA) were used. (**C,D**) Efficiency of Pygo2 knockdown by shRNAs. (**E**–**H**) Effects of Pygo2 knockdown on Wnt/β-catenin pathway component genes in human brain glioma U-87MG and U251 cell lines. Western blot analysis was used to check the expression of the target genes in U-87MG and U251 cell lines, with β-actin used as a control. Arrows (**→**) indicate cropping lines and gels were obtained under the same experimental conditions. (**E,F**) QRT-PCR analysis used to determine the mRNA expression (**G**,**H**). **P < 0.01 and ***P < 0.001.

Previous studies have revealed that Pygo2 acts as a core component of the Wingless/Wnt signaling pathway in Drosophila^23^,^24^. Pygo2 binds with other proteins such as Armadillo/β-catenin, Legless/Bcl9 and Pan/Tcf to form a functional complex critical in transcriptional activation^25^. To investigate roles of Pygo2 protein in the β-catenin pathway in glioma, we performed TOP/FOP flash analysis using the TOPflash reporter system in human brain glioma U-87MG and U251 cell lines. Three specific shRNAs with independent sequences against Pygo2 mRNA were designed to avoid possible off-target effects produced by shRNA. We found that the transfected Pygo2 shRNAs knock down the expression of Pygo2 mRNA in human brain glioma U-87MG and U251 cell lines, while the control scr-shRNA had no effect (Fig. 4C,D). We also found that the TOP flash activity which dictates the β-catenin pathway transcriptional activity is significantly decreased any of the three Pygo2 shRNAs (Fig. 4A,B).

To further confirm the inhibition of the Wnt/β-catenin pathway by Pygo2 knockdown, the mRNA and protein expression of the Wnt/β-catenin pathway downstream targets such as Cyclin D1, C-myc, Cyclin A, CD44, and β-actin (control) was checked. We found that the protein and mRNA levels of Cyclin D1, C-myc, Cyclin A and CD44 are both decreased by Pygo2 shRNA3 (Fig. 4E–H). Overall, these data revealed that Pygo2 also plays important regulatory roles in the Wnt/β-catenin pathway in human brain glioma cell lines.

### Pygo2 knockdown reduces the H3K4me3 protein expression

**Figure 5:**
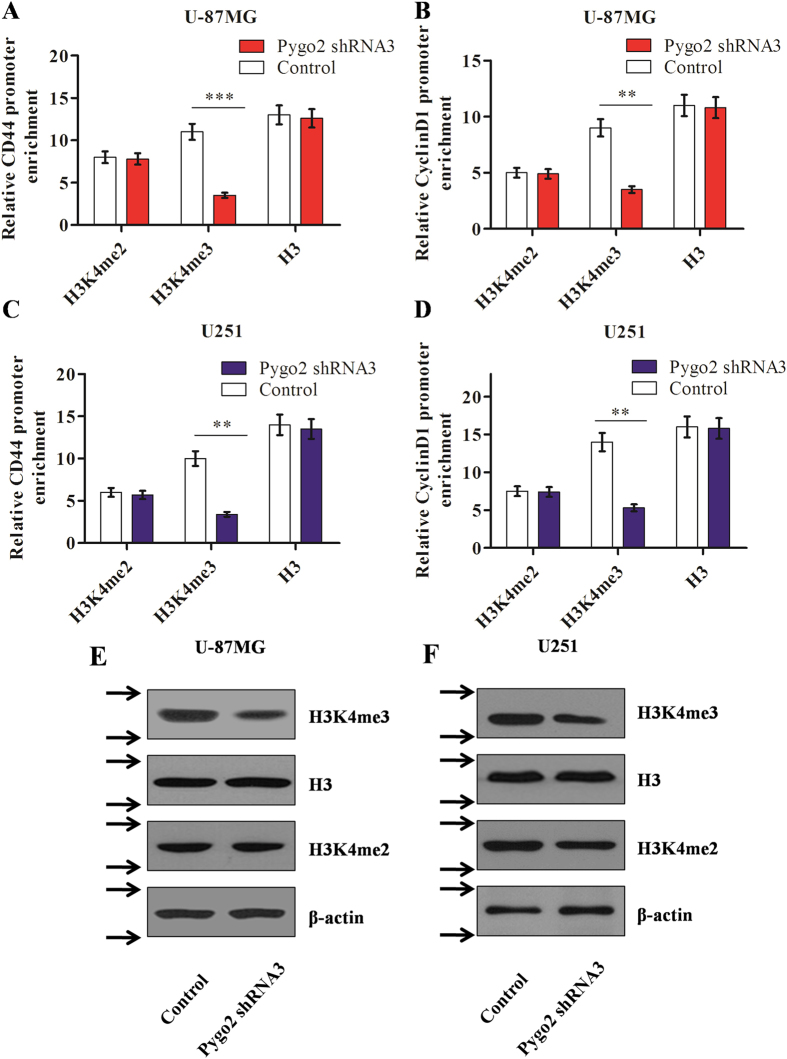
Effects of Pygo2 knockdown on H3K4me2 and -3 protein expression. (**A–D**) Effects of Pygo2 knockdown by shRNAs on the expressed of H3K4me2 and -3, present on the promoters of CD44 and CyclinD1, in human brain glioma U-87MG (**A**) and U251 (**B**) cell lines. The enrichment of H3K4me2/3 was measured by CHIP. Histone H3 was used as a negative control. (**E,F**) Effects of Pygo2 knockdown by shRNAs on the total protein expression of H3K4me2 and -3 in human brain glioma U-87MG (**E**) and U251 (**F**) cell lines. The protein levels were measured by western blot assay, with histone H3 used as a negative control. Arrows (**→**) indicate cropping lines and gels were obtained under the same experimental conditions. **P < 0.01 and ***P < 0.001.

Histone H3K4me3, modified in mammals by conserved COMPASS proteins, was generally considered a hallmark of transcriptional activation. And as previous studies have revealed, human Pygo2 protein could stimulate the transcriptional activation by directly binds H3K4me2 and H3K4me3^26^,^27^, but could Pygo2 affect the H3K4me2/3 protein expression, it was unclear. We therefore wanted to investigate whether and how shRNA knockdown of Pygo2 affects the protein levels of H3K4me2/3. A chromatin immunoprecipitation (CHIP) assay was performed to examine the enrichment of H3K4me2/3 on the Wnt/β-catenin pathway target gene promoters in human glioma U-87MG and U251 cell lines. We indeed found that H3K4me2/3 are present on the promoter of the CD44 and CyclinD1, and that the amount of H3K4me3, but not H3K4me2, bound to the CD44 and CyclinD1 gene promoters is decreased following knockdown by Pygo2 shRNA3 (Fig. 5A–D). Western blot assay was also used to investigate the global protein level of H3K4me2 and H3K4me3. It was revealed that the global protein level of H3K4me3, but not that of H3K4me2 and histone H, is decreased in the presence of Pygo2 shRNA3 (Fig. 5E,F). Collectively, these data suggest that Pygo2 not only binds to the H3K4me2/3 but also regulates the H3K4me3 level on the promoters of Wnt/β-catenin pathway target genes while it has no effect on H3K4me2.

### Pygo2 knockdown inhibits the recruitment of histone methyltransferase complex to the Wnt target genes promoters

**Figure 6:**
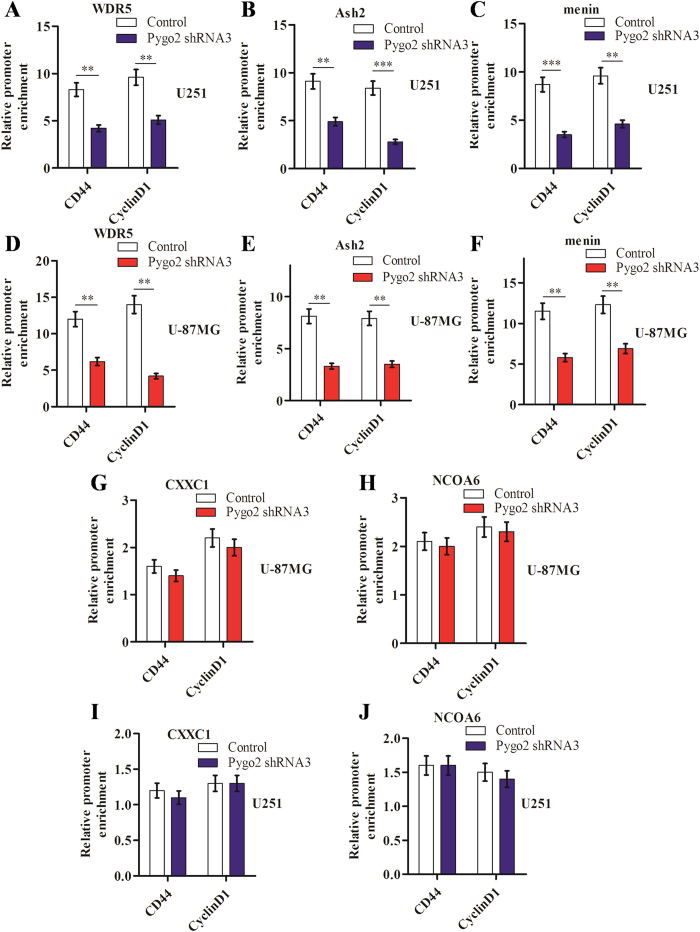
Effects of Pygo2 knockdown by shRNAs on the recruitment of histone methyltransferase complex to the Wnt target genes promoters. The core components of COMPASS-like complex, WDR5 and Ash2 on the promoters of Wnt/β-catenin pathway target genes in human brain glioma U251 (**A**,**B**) and U-87MG (**D**,**E**) cell lines were examined. The specific subunit of the MLL1/MLL2 histone methyltransferase (HMT) complex, menin (**C**,**F**), and specific subunits of SET-1 and MLL3/MLL4, CXXC1 (**G**,**I**) and NCOA6 (**H**,**J**), were also examined similarly. *P < 0.05 and **P < 0.01.

We next investigated a possible link between Pygo2 and KMTs. Histone H3 methylation is known to be catalyzed by lysine methyl transferases (KMTs). Eg, KMT2 trimethylates histone H3K4 and is part of the highly conserved COMPASS complex. Thus we examined whether any COMPASS or COMPASS-like complex is present on the promoter of pathway target genes and is regulated by Pygo2. For this we performed further CHIP assays in human brain glioma cell lines. Indeed we found that WDR5 and Ash2, two core components of the COMPASS complex^28^,^29^, are interact with the CD44 and CyclinD1 promoters, and down-regulated by Pygo2 knockdown (Fig. 6A,B,D,E). We also examined the expression of menin, the product of the multiple endocrine neoplasia type 1 (MEN1) tumor suppressor gene, which is known to be associated with MLL1/MLL2 histone methyltransferase (HMT) complexes, CXCC1 and NOCA6, specific subunits of SET and MLL3/MLL4, respectively. We found that menin expression, but not that of CXCC1 or NOCA6, diminished by Pygo2 knockdown (Fig. 6C,F,G–J). In summary, our data suggest that Pygo2 recruits the MLL1/MLL2 histone methyltransferase complex to Wnt/β-catenin target gene promoters to activate these genes through modifying histone H3 Lys4 trimethylation.

### Pygo2 knockdown inhibits glioma cell proliferation, migration and invasion

**Figure 7:**
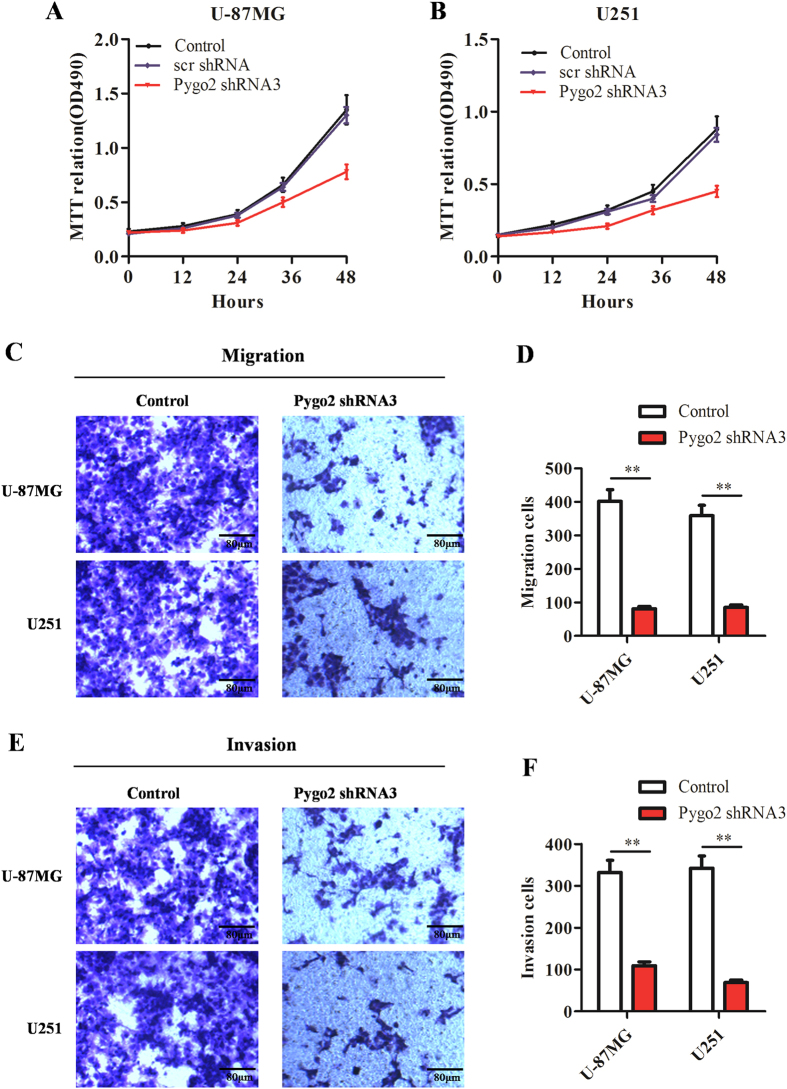
Effects of Pygo2 shRNA knockdown on glioma cell proliferation, migration and invasion. (**A,B**) Cell proliferation analysis by MTT assays using U-251 and U-87MG glioma cells with or without Pygo2 knockdown. Shown data were from three independent measurements (mean ± SD). (**C–F**) Effects of Pygo2 on migration and invasion of U-87MG and U251 glioma cells. Representative micrographs and statistical data showing the effects of Pygo2 knockdown on cell migration (**C**,**D**) and invasion (**E**,**F**). Data were presented as mean ± SD. Two-tailed Student’s t-test was used. *P < 0.05 and **P < 0.01.

We next examined the effects of Pygo2 on malignant phenotypes in glioma cells, including proliferation, migration and invasion. For this we performed MTT assays and found that the proliferation of glioma U251 and U-87MG cells is significantly decreased by Pygo2 knockdown (Fig. 7A,B).

We next utilized transwell assays to determine the effect of Pygo2 on cell migration. For this we incubated glioma cells in transwell chambers for 6 hours before counting cells that have crossed the insert (see Methods). We found that, compared with control U-87MG and U251 cells, those transfected with Pygo2 shRNA3 show significantly decreased migratory ability (Fig. 7C,D). To examine the effect of Pygo2 on cell invasion, we cultured U-87MG and U251 cells transfected with Pygo2 shRNA3 in transwell chambers pre-coated with matrigel for 8 hours before measurements. We found that Pygo2 knockdown significantly decreases the ability of cells to cross the matrigel (Fig. 7E,F). Taken together, our data demonstrated that Pygo2 knockdown significantly inhibits glioma cell proliferation, invasion and migration.

## Discussion

Previous studies have revealed that Pygo2 is over-expressed in several types of tumor and plays important roles in tumor progression^30^,^31^. Here we demonstrated that the mRNA and protein of Pygo2 are both elevated in human glioma tissues and cell lines compared to normal tissues and paraneoplastic tissues. Furthermore, we showed that Pygo2 expression strongly correlates with clinical and pathological features of glioma. In fact, the expression of Pygo2 was significantly higher in ttissues from the elderly and more severe (classified by WHO) glioma patients. By use of Kaplan–Meier analysis and survival curve, we also revealed correlation of Pygo2 abnormal expression with poor prognosis in human brain glioma patients.

We also examined the prognostic value for tissues with high Pygo2 expression using a Cox proportional hazard model. Univariate analysis identified stage clinically classified by WHO and found that Pygo2 expression is a poor prognosticator for overall patient’s survival (P = 0.035), while other features including age and gender are not significantly associated with overall patient’s survival. Patients with high Pygo2 expression correlated with a higher risk of human brain glioma progression compared to patients with low Pygo2 expression (P < 0.001). Furthermore, a multivariate analysis was performed and found that high Pygo2 expression is an independent feature for poor overall survival in human glioma patients (HR = 5.798, 95% CI = 1.137–29.582, P = 0.035) (Table 2). The abnormal expression of Pygo2 has a high risk to brain glioma oncogenesis.

By immunohistochemical assays and the immunofluorescent staining in human brain glioma tissue and cell lines we also provided further evidence to show that Pygo2 is a nuclear protein, consistent with previous reports about the nuclear localization of Pygo2 cancerous lung tissues^18^,^32^. However, we also found that Pygo2 is present in the cytoplasm in which its role remains to be determined.

One function of mammalian Pygo2 is that Pygo2 acts downstream of β-catenin to efficiently activate Wg/Wnt target gene transcription according to reported epistatic and molecular analyses^33^. And this is achieved by tethering to the β-catenin transcriptional complex via the interaction of its PHD domain with HD1 domain of protein Lgs/Bcl9, and using its conserved N-terminal homology domain (NHD)^34^. Previous studies have also provided strong evidence for the PHD domain of mammalian Pygo2 proteins to directly bind H3K4me2 and -3^25^,^26^. In the present study we discovered a novel function of Pygo2 as being able to regulate H3K4 trimethylation through specifically up-regulating the H3K4me3, but not the H3K4me2, expression.

It was reported Pygo2 proteins stimulated speculations concerning its potential role in the transcriptional regulatory function of Pygo2. For example, mediates the binding to Lgs/Bcl9 as well as with the chromatin at Wnt target loci and recruits/stabilizes the interaction of transcriptional co-factors with β-catenin^22^. Our result also demonstrated that in human glioma brain cell lines Pygo2 plays a crucial role in activating the transcription of Wnt genes and regulating the recruitment of f WDR5 and Ash2, two core components of the COMPASS complex, and menin, a specific component of the MLL1/MLL2 histone methyltransferase (HMT) complex, to the Wnt pathway target gene promoter loci. In addition, it was discovered that the latency associated nuclear antigen (LANA) interacts with Pygo2 protein by proteomic screen^35^. As we know, the Kaposi’s sarcoma-associated herpesvirus (KSHV) LANA functions in latently infected cells as an essential participant in KSHV genome replication and as a driver of deregulated cell growth. It is thus interesting to determine in the future whether Pygo2 interacts with LANA and is involved in virus latent and cracking progress.

In eukaryotes, covalent modifications of histone tails play a key role in regulating chromatin remoulding and controlling transcriptional activity^23^. Furthermore, H3 trimethylated at lysine 4 (H3K4me3) is associated with chromation activation and gene expression^36^,^37^. From the result of our study, the global level of H3K4me3 was decreased, we also consider another gene expression could be activated just not only the Wnt pathways, which will be subjects of future studies.

## Materials and Methods

### Clinical specimens

A total of 209 human glioma tissues,13 peritumoral tissues and 9 adjacent normal tissues were collected from human glioma patients in the First Affiliated Hospital of Anhui Medical University (Anhui, China), Fourth Hospital of Hebei Medical University (Tumor Hospital of Hebei Province, Hebei, China), Hubei General Hospital (Renmin Hospital of Wuhan University, Hubei, China), and Zhongnan Hospital of Wuhan University (Hubei, China) between 2004 and 2013 during surgery and made into paraffin sections (4 μm), No enrolled patients underwent radiation or chemotherapy prior to surgery. All specimen collections and studies thereof were approved by the Ethics Committee of the source hospitals and all patients provided the written informed consent for this study. All experiments were performed in accordance with principles expressed in the Declaration of Helsinki or other relevant guidelines and regulations.

### Cell culture and transfection

The human brain glioma U-87MG and U251 cell lines were purchased from the Cell Center of Institute of Biochemistry and Cell Biology, Chinese Academy of Sciences (Shanghai, China). Cells were cultured in Dulbecco’s modified Eagle’s medium (DMEM) (Gibco, USA) supplemented with 10% fetal bovine serum (Gibco, USA), 100 U/ml penicillin G and 100 μg/ml streptomycin at 37 °C in a humidified incubator containing 5% CO_2_. Human microglia cells (Cat#1900, ScienCell, USA) were used as control. The Lipofectamine 2000 Transfection Reagent (Invitrogen, Carlsbad, CA, USA) was used to transfect the U251 and U-87MG cell lines according to the manufacturer’s instructions. U251 and U-87MG cells were treated with Lipofectamine 2000 followed by transfection with the Pygo2 shRNA1, Pygo2 shRNA2, and Pygo2 shRNA3 shRNA-expressing recombinant plasmids and scr-shRNA, which was used as a negative control.

### RNA extraction and reverse-transcription quantitative PCR (qRT-PCR)

RNAs were extracted using Ambion® RNA extraction kit (Cat# 10928-034, Life Technologies, USA) according to the manufacturer’s instructions. Then, RNAs were than reverse transcribed and amplified by qRT-PCR using the SuperScript® One-Step RT-PCR System with Platinum® Taq DNA Polymerase (Life Technologies, USA) on a 7500 Real Time PCR System (Applied Biosystems, Mannheim, Germany). The annealing and extension steps were separated as follows (three-step cycling): Step 1, 1 cycle at 45–55 °C for 20 minutes plus 94 °C for 2 minutes; step 2, 35 cycles of denaturing at 94 °C for 15 seconds, annealing at 55–60 °C for 30 seconds and extending at 68–72 °C for 1 minute/kb.

### Western blotting and immunofluorescence

Total proteins from lysates of tissues and U-87MG and U251 cell lysates were extracted by re-suspending the cell pellets in RIPA buffer (150 mM NaCl, 50 mM Tris (pH 7.4) and 1% Triton X-100). Approximately 55 μg of total protein per sample was separated by SDS-PAGE and then transferred to a nitrocellulose membrane. Western blot analyses were performed with polyclonal antibodies against Pygo2, CyclinD1, CyclinA, C-myc, CD44 (Santa Cruz Biotechnology, USA), H3K4me3, anti-H3K4me2, and H3 (Sigma, USA), monoclonal β-actin antibody as control (Sigma, USA). Immunofluorescence was performed using a standard procedure with primary antibodies against Pygo2 (Santa Cruz Biotechnology, USA) and GFAP (Sigma, USA).

### Immunohistochemistry

Paraffin sections were deparaffinized successively in 100% xylene, 95% alcohol, 90% alcohol, 80% alcohol and 70% alcohol and then rehydrated for 10 minutes. Then, hydrogen peroxide (0.3% v/v) was applied to block endogenous peroxide activity and the samples were microwave heated in 15 μM citrate buffer (pH 6.0) for 3 minutes to expose the antigen site. Paraffin sections were then incubated with normal goat serum to reduce non-specific antibody binding. Then, the glioma tissue sections were incubated with a Pygo2 polyclonal antibody (1:500 dilution, Santa Cruz Biotechnology). Rabbit immunoglobulin (1:500 dilution) was used as a negative control. Antibody staining was performed by overnight incubation at 4 °C with gentle shaking. Then, the samples were incubated with the secondary biotinylated goat anti-rabbit serum immunoglobulin G (IgG) antibody at 37 °C for 30 minutes. After washing, the paraffin sections were incubated with streptavidin–avidin-conjugated horseradish peroxidase for 30 minutes. Counterstaining with hematoxylin was carried out for 30 minutes, and the paraffin sections were dehydrated in ethanol prior to mounting. Estimation of Pygo2 staining in tissue sections was performed according to the immunohistochemical assessment used (Popadiuk *et al.*). Briefly, the Pygo2 staining intensity was scored as: − (no signal), +(weak), ++ (moderate), or +++ (strong).

### TOP/FOP flash luciferase assay

Luciferase activity was evaluated using the Dual Luciferase Reporter Assay System (Promega, USA). The human brain glioma U-87MG and U251 cell lines were transfected with TOP-flash or FOP flash plasmid (Promega, USA) along with an internal Renilla control plasmid (Promega, USA) according to the manufacturer’s protocol.

### Chromatin immunoprecipitation assay

U-87MG or U251 cells (~5.5 × 10^6^ cells) were cross-linked by the addition of 1% formaldehyde in PBS and incubated for 10 minutes at 37 °C. The cells were washed twice in PBS and lysed in SDS lysis buffer (1% SDS, 40 mmol/L Tris (pH 7.9), 15 mmol/L EDTA, 1.5 mmol/L DTT, and protease inhibitors). The lysate was sonicated for 15 seconds in a Bioruptor 14 times. Soluble material was purified and diluted 10-fold in dilution buffer (100 mmol/L NaCl, 1.5 mmol/L DTT, 25 mmol/L Tris-HCl (pH 7.9), 2.5 mmol/L EDTA, 2% Triton X-100, and protease inhibitors) and incubated with the appropriate antibody (polyclonal antibodies against WDR5, Ash2, menin, CXXC1 or NCOA6) or control rabbit IgG overnight at 4 °C, and then incubated with 60μl of Protein A Agarose/Salmon Sperm DNA under reverse rotation at 4 °C for 2 hours. The beads were washed four times with wash buffer and once in TE buffer. The beads were resuspended in elution buffer (100 μl 1 M NaHCO3 and 100 μl 10% SDS) and reverse DNA/protein cross-linked at 65 °C overnight. Elution buffer (2 μl 10 mg/ml Proteinase K, 10 μl 0.5 M EDTA, 20 μl 1 M Tris (PH 6.5), and 1 μl RNaseA (MBI)) was added for 2 hours at 45 °C. Binding to the DNA promoter was assessed by quantitative PCR using the primers listed in Table 3.

### MTT assay

Cells (1 × 10^5^ cells/well) were seeded into 6-well plates. The cell proliferation was examined at 12, 24, 36 and 48 hours after transfection. Cells were stained at the indicated time points with 100 μl sterile MTT dye (0.5 mg/ml, Sigma, USA) for 4 hours at 37 °C, followed by removal of the culture medium and the addition of 150 μl DMSO (Sigma). The number of viable cells was assessed by measurement of the absorbance at 490 nm. All experiments were performed in triplicates.

### Cell migration and invasion assays

For cell migration assays, 1 × 10^4^ cells in 100 μl DMEM without fetal bovine serum were seeded on a fibronectin-coated polycarbonate membrane insert in a transwell chamber (Costar Corning, USA). In the lower chamber, 500 μl DMEM with 10% fetal bovine serum was added as chemo-attractant. After the cells were incubated for 6 hours at 37 °C with 5% CO2, the insert was washed with phosphate buffered saline, and cells on the top surface of the insert were removed with a cotton swab. Cells adhering to the lower surface were fixed with methanol, stained with crystal violet solution, and quantified with ImageJ software. All assays were independently repeated at least three times. The procedure for cell invasion assays was similar to that of cell migration assays, except that the transwell membranes were precoated with 24 μg/μl matrigel (R&D Systems Inc., USA) and that cells were incubated for 8 hours at 37 °C with 5% CO2. Cells adhering to the lower surface were counted the same way as in cell migration assays.

### Statistical analysis

Statistical analysis was performed using SPSS 13.0 for Windows. The χ2 test was used to evaluate the relationship between Pygo2 expression profiles and clinicopathological features. Receiver operating characteristic (ROC) analysis was performed to determine the diagnostic performance of Pygo2 mRNA expression levels in distinguishing patients with glioma from healthy control subjects. Sensitivity against 100% specificity was plotted for each cutoff threshold; the area under the curve (AUC) values that reflect the probability of correctly identifying glioma patients from control subjects were computed. Kaplan–Meier analysis was used to evaluate the probability of patient survival, and distributions were evaluated using the long-rank test. The Cox proportional hazard models were used to calculate hazard ratios and identify factors affecting the survival. Differences in characteristics or values were examined by the χ2 and Fisher’s exact tests. Statistical significance was based on a P-value of 0.05.

## Additional Information

**How to cite this article**: Zhou, C. *et al.* Pygo2 functions as a prognostic factor for glioma due to its up-regulation of H3K4me3 and promotion of MLL1/MLL2 complex recruitment. *Sci. Rep.* **6**, 22066; doi: 10.1038/srep22066 (2016).

## Supplementary Material

Supplementary Information

## Figures and Tables

**Table 1:** Correlation between Pygo2 expression and clinicopathological features of human glioma tissues.

Clinicopathological features	n	Pygo2		P value^a^
		Over expression (%)	Nomal expression (%)	
Age				
≤50 years	56	31 (55.36)	25 (44.64)	0.001*
>50 years	153	121 (79.08)	32 (20.92)	
Gender				
Male	137	102 (74.45)	35 (25.55)	0.440
Female	72	50 (69.44)	22 (30.56)	
Astrocytoma				
Anaplastic astrocytoma	63	46 (73.02)	17 (26.98)	0.984
Diffuse astrocytoma	65	49 (75.38)	16 (24.62)	
Yellow astrocytoma	4	3 (75.00)	1 (25.00)	
WHO phase				
I	26	11 (42.31)	15 (57.69)	0.000*
II	77	46 (59.74)	31 (40.26)	
III	59	48 (81.36)	11 (18.64)	
IV	47	44 (93.62)	3 (6.38)	

^a^Statistical significance was determined using Fisher’s exact test.

^*^Significant relation of clinicopathological features with glioma.

**Table 2:** Variables in the COX multivariate analysis.

Variables	HR	P value	95% CI
Pygo2 expression	5.798	0.035*	1.137–29.582
Age	2.036	0.126	0.818–5.066
Gender	2.304	0.08	0.906–5.860
WHO phase	0.183	0.003*	0.061–0.551

CI: confidence interval; HR: hazard ratio.

^*^significant relation of clinicopathologic features with progression survival.

**Table 3:** Primers for Pygo2 shRNAs, qRT-PCR, and CHIP.

Name		Sequence	Length
Pygo2 shRNAs			
Pygo2 shRNA1	F	5′-CACCGCCGGTCTGCAAATGAAGAGTTCAAGAGACTCTTCATTTGCAGACCGGC-3′	53 bp
	R	5′-AAAAGCCGGTCTGCAAATGAAGAGTCTCTTGAACTCTTCATTTGCAGACCGGC-3′	53 bp
Pygo2 shRNA2	F	5′-CACCGAAGCGAAGGAAGTCAAATACTCAAGAGGTATTTGACTTCCTTCGCTTC-3′	53 bp
	R	5′-AAAAGAAGCGAAGGAAGTCAAATACCTCTTGAGTATTTGACTTCCTTCGCTTC-3′	53 bp
Pygo2 shRNA3	F	5′-CACCAGGCCGGTCTGCAAATGAAGATCAAGAGTCTTCATTTGCAGACCGGCC-3′	52 bp
	R	5′-AAAAGGCCGGTCTGCAAATGAAGACTCTTGATCTTCATTTGCAGACCGGCCT-3′	52 bp
qRT-PCR			
Pygo2	F	5′-AGAAAAGAAGCGAAGGAAGTCAAA-3′	24 bp
	R	5′-GGTGATCCACCATGGGAGTT-3′	20 bp
	P	5′ FAM-CTCACATCTGACGGAGTTTGCACCACC-BHQ 3′	27 bp
CyclinD1	F	5′- GCGTGTAGCTATGGAAGTTGCA-3′	22 bp
	R	5′- CATCCCGAATGAGAGTCCTACAG-3′	23 bp
	P	5′ FAM-CTTACGTGCCACCACGGCGTTGT-BHQ 3′	23 bp
C-myc	F	5′-ATCTCACAGTGACCAACCCAAA-3′	22 bp
	R	5′- TCGGTCACGGAGCCAATC-3′	18 bp
	P	5′ FAM-CGTGAACGAGAAGTCCTGCAACTGCC-BHQ 3′	26 bp
CyclinA	F	5′- ATGAAGAAACAGCCAGACATCAC-3′	23 bp
	R	5′- CTCTCAGCACTGACATGGAAG-3′	21 bp
	P	5′- TGAGAGCTATCCTCGTGGACTGGTTAGTTGA-BHQ 3′	31 bp
CD44	F	5′- AGTCGTCAGAAACTCCAGACCAGT-3′	24 bp
	R	5′- CCAGCTCCCTGTAATGGTTATGTT-3′	24 bp
	P	5′- CAGCTGATGAGACAAGGAACCTGCAGAA-BHQ 3′	28 bp
CHIP			
CyclinD1 promoter	F	5′-CACCCCCAACAAAACCAATTAG-3′	22 bp
	R	5′-ACGTTACTGTTGTTAAGCAAAGATCA-3′	26 bp
CD44 promoter	F	5′- CAAACACACACATTTTGCAGCATAT-3′	25 bp
	R	5′- CCCTCTGGTACGGAATAAAACTCA-3′	24 bp

## References

[b1] Alexandra K. & Walter B. Wnt signalling and its impact on development and cancer. Nat Rev Cancer. 8, 387–98 (2008). 18432252 10.1038/nrc2389

[b2] Dan N. R. *et al.* LRP5/6 directly bind to Frizzled and prevent Frizzled-regulated tumour metastasis. Nat Commun. 6, 6906 (2015). 25902418 10.1038/ncomms7906

[b3] Fukuzawa R., Anaka M. R., Weeks J. R., Morison M. I. & Reeve A. E. Canonical WNT signalling determines lineage specificity in Wilms tumour. Oncogene. 28, 1063–75 (2009). 19137020 10.1038/onc.2008.455

[b4] Clevers H. Wnt/β-Catenin Signaling in Development and Disease. Cell. 127, 469–80 (2006). 17081971 10.1016/j.cell.2006.10.018

[b5] Zhang J. *et al.* The Wnt/β-catenin pathway drives increased cyclin D1 levels in lymph node metastasis in papillary thyroid cancer. Hum Pathol. 43, 1044–50 (2012). 22204713 10.1016/j.humpath.2011.08.013

[b6] Zhang S. *et al.* Wnt/β-catenin signaling pathway upregulates c-Myc expression to promote cell proliferation of P19 teratocarcinoma cells. Anat Rec. 295, 2104–13(2012). 10.1002/ar.22592 22976998

[b7] Suzuki A., Scruggs A. & Iwata J. The temporal specific role of WNT/β-catenin signaling during myogenesis. J Nat Sci. 1, e143 (2015). 26176019 PMC4499510

[b8] Wielenga V. J. *et al.* Expression of CD44 in Apc and Tcf mutant mice implies regulation by the WNT pathway. Am J Pathol. 154, 515–23 (1999). 10027409 10.1016/S0002-9440(10)65297-2 PMC1850011

[b9] Li B., Mackay D. R., Ma J. & Dai X. Cloning and developmental expression of mouse pygopus 2, a putative Wnt signaling component. Genomics. 84, 398–405 (2004). 15234002 10.1016/j.ygeno.2004.04.007 PMC2893388

[b10] Schwab K. R. *et al.* Pygo1 and Pygo2 roles in Wnt signaling in mammalian kidney development. BMC Biol. 5, 15 (2007). 17425782 10.1186/1741-7007-5-15 PMC1858683

[b11] Popadiuk C. M. *et al.* Antisense suppression of pygopus 2 results in growth arrest of epithelial ovarian cancer. Clin Cancer Res. 12 (7 Pt 1), 2216–23 (2006). 16609037 10.1158/1078-0432.CCR-05-2433

[b12] Andrews P. G., Lake B. B., Popadiuk C. & Kao K. R. Requirement of pygopus 2 in breast cancer. Int J Oncol. 30, 357–63 (2007). 17203217

[b13] Wang Z. X. *et al.* Decreased pygopus 2 expression suppresses glioblastoma U251 cell growth. J Neurooncol. 100, 31–41 (2010). 20204459 10.1007/s11060-010-0144-6

[b14] Nakamura Y. *et al.* Crystal structure analysis of the PHD domain of the transcription co-activator Pygopus. J Mol Biol. 370, 80–92 (2007). 17499269 10.1016/j.jmb.2007.04.037

[b15] Dong S. *et al.* Wnt5a Promotes Cytokines Production and Cell Proliferation in Human Hepatic Stellate Cells Independent of Canonical Wnt Pathway. Clin Lab. 61, 537–47 (2015). 26118187 10.7754/clin.lab.2014.141127

[b16] Zhang L., Bahety P. & Ee P. L. Wnt co-receptor LRP5/6 over-expression confers protection against hydrogen peroxide-induced neurotoxicity and reduces tau phosphorylation in SH-SY5Y cells. Neurochem Int. 87, 13–21 (2015). 25959626 10.1016/j.neuint.2015.05.001

[b17] Santos R. H. *et al.* Active genes are tri-methylated at K4 of histone H3. Nature. 419, 407–11 (2002). 12353038 10.1038/nature01080

[b18] Mohan M., Lin C., Guest E. & Shilatifard A. Licensed to elongate: a molecular mechanism for MLL-based leukaemogenesis. Nat Rev Cancer. 10, 721–8 (2010). 20844554 10.1038/nrc2915

[b19] Liu Y. *et al.* Abnormal expression of Pygopus 2 correlates with a malignant phenotype in human lung cancer. BMC Cancer. 13, 346 (2013). 23865714 10.1186/1471-2407-13-346 PMC3718726

[b20] Andrews P. G. *et al.* Evidence of a novel role for Pygopus in rRNA transcription. Biochem J. 453, 61–70 (2013). 23517060 10.1042/BJ20121667

[b21] Andrews P. G., Kennedy M. W., Popadiuk C. M. & Kao K. P. Oncogenic activation of the human Pygopus2 promoter by E74-like factor-1. Mol Cancer Res. 6, 259–66 (2008). 18314487 10.1158/1541-7786.MCR-07-0068

[b22] Townsley F. M., Cliffe A. & Bienz M. Pygopus and Legless target Armadillo/β-catenin to the nucleus to enable its transcriptional co-activator function. Nat Cell Biol. 6, 626–33 (2004). 15208637 10.1038/ncb1141

[b23] Parker D. S., Jemison J. & Cadigan K. M. Pygopus, a nuclear PHD-finger protein required for Wingless signaling in Drosophila. Development. 129, 2565–76 (2002). 12015286 10.1242/dev.129.11.2565

[b24] Kramps T. *et al.* Wnt/wingless signaling requires BCL9/legless-mediated recruitment of pygopus to the nuclear β-catenin-TCF complex. Cell. 109, 47–60 (2002). 11955446 10.1016/s0092-8674(02)00679-7

[b25] Mosimann C., Hausmanng G. & Basler K. Parafibromin/Hyrax activates Wnt/Wg target gene transcription by direct association with β-catenin/Armadillo. Cell. 125, 327–41 (2006). 16630820 10.1016/j.cell.2006.01.053

[b26] Fiedler M. *et al.* Decoding of methylated histone H3 tail by the Pygo-BCL9 Wnt signaling complex. Mol Cell. 30, 507–18 (2008). 18498752 10.1016/j.molcel.2008.03.011 PMC2726290

[b27] Gu B. *et al.* Pygo2 expands mammary progenitor cells by facilitating histone H3 K4 methylation. J Cell Biol. 185, 811–26 (2009). 19487454 10.1083/jcb.200810133 PMC2711593

[b28] Wysocka J. *et al.* WDR5 associates with histone H3 methylated at K4 and is essential for H3 K4 methylation and vertebrate development. Cell. 121, 859–72(2005). 15960974 10.1016/j.cell.2005.03.036

[b29] Steward M. M. *et al.* Molecular regulation of H3K4 trimethylation by ASH2L, a shared subunit of MLL complexes. Nat Struct Mol Biol. 13, 852–4 (2006). 16892064 10.1038/nsmb1131

[b30] Hughes C. M. *et al.* Menin associates with a trithorax family histone methyltransferase complex and with the hoxc8 locus. Mol Cell. 13, 587–97 (2004). 14992727 10.1016/s1097-2765(04)00081-4

[b31] Yokoyama A. *et al.* Leukemia proto-oncoprotein MLL forms a SET1-like histone methyltransferase complex with menin to regulate Hox gene expression. Mol Cell Biol. 24, 5639–49 (2004). 15199122 10.1128/MCB.24.13.5639-5649.2004 PMC480881

[b32] Moghbeli M. *et al.* Association of PYGO2 and EGFR in esophageal squamous cell carcinoma. Med Oncol. 30, 516 (2013). 23456637 10.1007/s12032-013-0516-9

[b33] Mosimann C., Hausmann G. & Basler K. β-catenin hits chromatin: regulation of Wnt target gene activation. Nat Rev Mol Cell Biol. 10, 276–86 (2009). 19305417 10.1038/nrm2654

[b34] Hoffmans R., Stadeli R. & Basler K. Pygopus and legless provide essential transcriptional coactivator functions to armadillo/β-catenin. Curr Biol. 15, 1207–11 (2005). 16005293 10.1016/j.cub.2005.05.054

[b35] Shamay M. *et al.* A protein array screen for Kaposi’s sarcoma-associated herpesvirus LANA interactors links LANA to TIP60, PP2A activity, and telomere shortening. J Virol. 86, 5179–91 (2012). 22379092 10.1128/JVI.00169-12 PMC3347335

[b36] Schneider R. *et al.* Histone H3 lysine 4 methylation patterns in higher eukaryotic genes. Nat Cell Biol. 6, 73–7 (2004). 14661024 10.1038/ncb1076

[b37] Briggs S. D. *et al.* Histone H3 lysine 4 methylation is mediated by Set1 and required for cell growth and rDNA silencing in Saccharomyces cerevisiae. Genes Dev. 15, 3286–95 (2001). 11751634 10.1101/gad.940201 PMC312847

