# Supplementary material for: Pygo2 functions as a prognostic factor for glioma due to its up-regulation of H3K4me3 and promotion of MLL1/MLL2 complex recruitment
Source: Sci Rep. 2016 Feb 23;6:22066. doi: 10.1038/srep22066 (PMC4763266; doi:10.1038/srep22066)
Supplement: Supplementary Information [file srep22066-s1.pdf]

Supplementary information

Pygo2 functions as a prognostic factor for glioma due to its up-regulation of H3K4me3 and promotion of MLL1/MLL2 complex recruitment

Cefan Zhou[1], Yi Zhang[1], Jun Dai[1], Mengzhou Zhou[1], Miao Liu[2], Yefu Wang[3], Xing-Zhen Chen[1,4], Jingfeng Tang[1]*

[1]Membrane Protein Disease and Cancer Research Center, Provincial Cooperative Innovation Center of Industrial Fermentation, College of Bioengineering, Hubei University of Technology, Wuhan, Hubei, 430068, China; [2]Neurology department, Renmin Hospital of Wuhan University, Wuhan, Hubei, 430060, China ; [3]The State Key Laboratory of Virology, College of Life Sciences, Wuhan University, Wuhan, Hubei, 430072, China; [4]Membrane Protein Disease Research Group, Department of Physiology, Faculty of Medicine and Dentistry, University of Alberta, Edmonton, AB, Canada.

*To whom correspondence may be addressed: Membrane Protein Disease and Cancer Research Center, College of Bioengineering, Hubei University of Technology, Wuhan 430068, China.

*Corresponding authors: Jingfeng Tang

E-Mail: Jingfeng_HUT@163.com

Tel.: +86-27-5972-6585; Fax: +86-27-5922-0119.

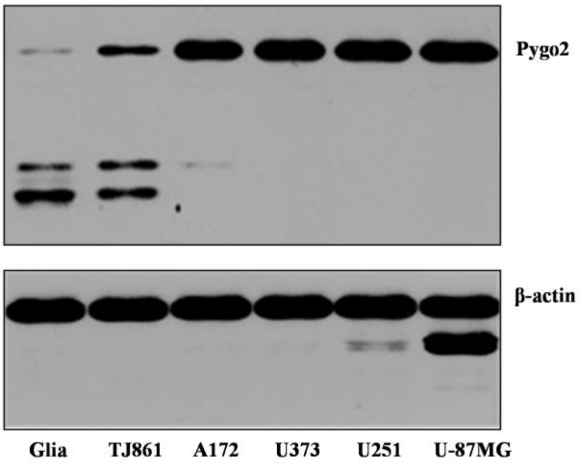

Supplementary Figure 1. Pygo2 protein expression in 5 glioma cell lines, TJ861, A172, U373, U251 and U-87MG, and normal human microglia cells (Glia, control). These are full-length blots corresponding to Fig. 1F in the main manuscript.
